# MEDICAL FITNESS IN WORKERS WITH PSYCHIATRIC DISORDERS

**DOI:** 10.1192/j.eurpsy.2023.1853

**Published:** 2023-07-19

**Authors:** L. Ben Afia, G. Bahri, I. Youssef, N. Mechergui, S. Ernez, D. Brahim, M. Mersni, N. Ladhari

**Affiliations:** Occupational medecine department, Charles Nicolle Hospital, Tunis, Tunisia

## Abstract

**Introduction:**

Mental health disorders are among the most burdensome health concerns in the world; it affects more than 970 million people in 2019. These disorders deteriorate all aspects of life, especially the professional field, impacting mainly physical capability, daily functioning, and productivity among the working-age population.

**Objectives:**

To study the socio-professional and medical characteristics of workers with psychiatric disorders and to assess the repercussions of these pathologies on work ability.

**Methods:**

A descriptive and retrospective study included all the medical files of workers with psychiatric disorders who were referred to the occupational medicine department at Charles Nicolle Hospital for a medical opinion of fitness for work during the period from January 1, 2014, to December 31, 2020.

**Results:**

The average age of the 224 cases collected was 41.74± [25-60 years] with a sex ratio of 0.67. The average professional seniority was 13.4 years±8.27 years. The most common occupational sectors were health (38.1%) and communication (20.2%). The patients were mainly suffering from either an anxiety-depressive disorder (36.6%) or psychosis (11.6%). These included 21 cases of bipolar disorder, 21 cases of schizophrenia, two cases of chronic hallucinatory psychosis and 3 cases of delusional psychosis. Forty-three patients were fit to continue working, 133 patients were fit with restrictions and twenty-six were unfitted to work.

A professional reclassification was recommended for 37 patients in positions with a lower mental load. One employee suffering from advanced schizophrenia was offered early retirement on grounds of disability. Permanent unfitness was pronounced in 11.6% of cases.

**Conclusions:**

The impact of mental disorders on cognitive abilities can be so significant as to result in temporary or permanent unfitness for work. However, the decision of medical fitness for work for the same psychiatric pathology may vary from one individual to another and from one workstation to another.

**Disclosure of Interest:**

None Declared

